# Effects of slightly acidic electrolyzed water on the quality and antioxidant capacity of fresh red waxy corn during postharvest cold storage

**DOI:** 10.3389/fpls.2024.1428394

**Published:** 2024-06-13

**Authors:** Chunfang Wang, Hongru Liu, Chenxia Liu, Yuzhen Wei, Juanzi Wang, Yi Zhang, Xiao Wang, Bingjie Chen, Weiqiang Yan, Yongjin Qiao

**Affiliations:** ^1^ Crop Breeding and Cultivation Research Institute, Shanghai Academy of Agricultural Sciences, Shanghai, China; ^2^ School of Information Engineering, Huzhou University, Huzhou, Zhejiang, China; ^3^ College of Food Science and Technology, Shanghai Ocean University, Shanghai, China; ^4^ Shanghai Shuneng Irradiation Technology Co., Ltd, Shanghai Academy of Agricultural Sciences, Shanghai, China

**Keywords:** fresh red waxy corn, slightly acidic electrolyzed water (SAEW), nutrition properties, antioxidant enzymes, cold storage

## Abstract

Fresh red waxy corn is consumed worldwide because of its unique flavor and rich nutrients, but it is susceptible to deterioration with a short shelf life. This study explored the effect of slightly acidic electrolyzed water (SAEW) treatment on the quality and antioxidant capacity of fresh red waxy corn during postharvest cold storage up to 40 d. The SAEW treatment exhibited lower weight loss, softer firmness, and higher total soluble solids (TSS) and moisture content than the control group. Correspondingly, the SAEW maintained the microstructure of endosperm cell wall and starch granules of fresh red waxy corn kernels well, contributing to good sensory quality. Furthermore, SAEW effectively reduced the accumulation of H_2_O_2_ content, elevated the O_2_^−^· scavenging ability, maintained higher CAT and APX activities, and decreased the decline of the flavonoids and anthocyanin during the storage. These results revealed that the SAEW treatment could be a promising preservation method to maintain higher-quality attributes and the antioxidant capacity of fresh red waxy corn during postharvest cold storage.

## 1 Introduction

Fresh waxy corn (*Zea mays* L. *sinensis Kulesh*) is widely grown and increasingly consumed as a fresh vegetable in East Asia and Southeast Asia, because of its chewy texture, tenderness, stickiness, enhanced nutrition, and unique flavor (Simla et al., 2010; Saikaew et al., 2018; Lim and Yi, 2019). It is rich in carbohydrates, phytochemicals, and micronutrients, and is becoming more popular as a healthy whole grain food (Chen et al., 2021). In 2023, the fresh corn market consumption in China is 57 billion ears, with the fresh waxy corn accounting for 64%. The market consumption continues to grow. The pigmented fresh waxy maize is preferred by consumers due to the health-enhancing effects of anthocyanin pigments and other secondary metabolites (Hu et al., 2020). However, fresh corn is prone to deterioration due to its high postharvest respiration rate and metabolic level, and the kernel desiccation and loss of taste occurred in 3 days when stored at room temperature (Meng et al., 2013; Wang et al., 2019). As the fresh waxy corn “Zhongnuo 1” is stored at 20°C for 4 d, the contents of sucrose, reducing sugars, and total soluble sugars reduced approximately threefold, while the firmness increased by 80% compared to 0 d, which severely influenced the sensory quality of the fresh waxy corn (Gong and Chen, 2013; Gong et al., 2018). The modified atmosphere packaging extended the shelf life of the fresh sweet corn to 5 d at ambient temperature (Liu et al., 2021). However, the extension time of the fresh corn was left much to the demand of the practical production. Quick cooling upon harvesting and refrigeration are effective preservation methods that could inhibit the respiration rate and delay the senescence rate, extending the shelf life (Techavuthiporn and Boonyaritthongchai, 2016). However, refrigeration also produced some adverse effects on quality. For instance, low temperatures would slow down enzymatic activities, affecting the nutritional quality and metabolic processes of stored produce. In addition, low temperatures induced physiological disorders, such as internal browning and uneven ripening, and altered cell structure. Novel postharvest technologies and environment-friendly preservation agents are necessary to evaluate the preservation effects on fresh waxy corn.

Electrolyzed water is produced by electrolysis of tap water and diluted NaCl or HCl solution with or without membrane in the electrolytic chamber (Hayta and Aday, 2015; Chen et al., 2020). Slightly acidic electrolyzed water (SAEW) is one type of electrolyzed water with a pH range from 5.0 to 6.5 (Rahman et al., 2016). SAEW has been proven to be biologically safe at a low available chlorine concentration (ACC) of 10–30 mg/L and authorized as a food additive in Japan since 2002 (Suzuki et al., 2005; Issa-Zacharia et al., 2011). In addition, SAEW is an environmentally safe and low-cost technique for postharvest preservation of fresh products (Sun et al., 2022; Zhang et al., 2023).

Recently, researchers started to explore the effect of SAEW on maintaining the postharvest properties of fruits and vegetables (Li et al., 2022; Zhang et al., 2023). SAEW had little adverse effect on total soluble solids (TSS), vitamin C, titratable acidity, weight loss and firmness of cherry tomatoes, strawberries, and carambola (Ding et al., 2015; Zhang et al., 2023). Meanwhile, the concentration of the ACC was important to influence the preservation effect. Electrolyzed water concentrations from 25 to 100 mg/L were efficient to maintain the freshness and quality of sweet cherry combined with passive atmosphere packaging storage, while above 200 mg/L, it had a negative impact (Hayta and Aday, 2015). The combination of SAEW with other technologies was also used to preserve the foods. SAEW combined with hydrogen-rich water (HRW) treatment maintained a higher storage quality of fresh-cut kiwifruit (Zhao et al., 2021). SAEW combined with ultrasonic treatment could reduce weight loss and improve color stability, anthocyanin content, and the activities of antioxidant enzymes of Chinese bayberry (Suo et al., 2023). These studies indicated that SAEW is a promising method to improve the storage properties and extend the shelf life of vegetables and fruits. However, the effects of SAEW on alleviating the quality deterioration of fresh waxy corn has not been reported.

Reactive oxygen species (ROS) are important signaling molecules and excess content damages the cell by oxidation of the DNA, proteins, and membrane, inducing the accelerated senescence and fruit decay (Zhou et al., 2014; Lin et al., 2015; Chu et al., 2018). In response to stress conditions and oxidation pressure, fruits and vegetables activated both their enzymatic and non-enzymatic protective mechanisms to clear the excess ROS (Xiang et al., 2020). Non-enzymatic antioxidant systems include antioxidative metabolites, such as flavonoids, anthocyanin, phenols, and ascorbic acid (Li et al., 2010). Catalase (CAT) and ascorbate peroxidase (APX) are antioxidant enzymes, which are crucial in scavenging ROS (Zhang et al., 2019). Therefore, this study investigated the effects of SAEW on the quality attributes of fresh red waxy corn “Huhongnuo No.1” during postharvest cold storage, and explored its influence on the enzymatic and non-enzymatic antioxidant metabolism processes.

## 2 Materials and methods

### 2.1 Materials and experiment treatments

Fresh red waxy corn “Huhongnuo No.1” at milk stage (22–25 d after pollination) were harvested from a commercial farm in Jiading district of Shanghai, China, and delivered to the laboratory in 2 h. The corn ears of uniform size, without disease, pests, and mechanical damage, were selected for the experiment. Based on the pre-experiments in our group, we confirmed the suitable concentration of the SAEW solution with ACC 30 mg L^−1^, pH 6.1. All the 160 corn ears of two groups were firstly precooled at 0–2°C for 10 h, and then randomly divided into two groups. One group was soaked with SAEW solution for 10 min, and the control group was soaked with distilled water. All the corn ears were dried with cool air within 30 min, and stored at 3 ± 1°C, RH 90 ± 5% for 40 d, covered with a polyethylene film. A total of 12 corn ears were sampled one time every 8 days and divided into three biological replicates randomly for evaluating the physiology and biochemistry properties. The kernels from the middle parts of each corn ear were sampled and shortly frozen with liquid nitrogen and stored at −80°C for the next use.

### 2.2 Weight loss and texture measurement

Twelve corn ears were prepared alone for determination of weight loss for both of the SAEW treatment and the control group. The percentage of weight loss was calculated as loss of weight compared to the initial weight (Patil et al., 2024).

The center region kernels of each corn ear were selected to determine the firmness with a texture analyzer (TA-XT Plus, Stable Micro System Ltd., Surrey, UK) equipped with a P2 probe. The penetration depth was 4 mm under a rate of 2 mm/s, and the firmness was expressed in newton (N).

### 2.3 Microstructure analysis

The cross-section microstructure of the fresh red corn kernels was observed with scanning electron microscopy (SEM) (TM 4000 plus, Hitachi, Tokyo, Japan) during cold storage. Frozen corn kernels were carefully cut in half at the endosperm crown and fixed with 2.5% glutaraldehyde solution for 24 h at 4°C. The samples were washed three times (each time for 15 min) with phosphate buffer (0.1 mol L^−1^, pH 7.4). After washing, they were dehydrated by graded ethanol (30%, 50%, 70%, 80%, and 90%) for 15 min respectively and then dehydrated three times by 100% alcohol for 15 min each time. Finally, the samples were frozen dried and coated with gold for scanning.

### 2.4 TSS, moisture, and soluble protein content

To guarantee the uniformity of the data, a total of 12 corn ears were divided into three biological replicates randomly and sampled. The kernels from the middle parts of each corn ear were sampled. Sampled fresh corn kernels were homogenized (HX-J118, AUX Group Co., Ltd., Ningbo, China) and used for the determination of TSS and moisture content. The homogenate was extruded with four layers of gauze. The filtrate was collected to assay the TSS with a Abbe refractometer (WYA-ZT, Shanghai INESA Physico-Optical Instrument Co., Ltd., Shanghai, China). The homogenate was used to determine the moisture content of corn kernels by a moisture dryer (V20S, Mettler Toledo, Switzerland) and the result was expressed in %. Three replicates were performed.

Frozen samples were ground using a cryogenic grinding machine (JX-FSTPRP-I, Shanghai Jingxin Industrial Development Co., Ltd., Shanghai, China) for the determination of soluble protein content and the following parameters. For soluble protein content, 1.0 g of sample was homogenized with 5.0 mL of distilled water and centrifuged at 4°C, 12,000 *g* for 20 min. The supernatant (1.0 mL) was mixed with 5.0 mL of Coomassie brilliant blue G-250 solution, stood for 2 min, and the absorbance value was measured at 595 nm. The results are expressed in mg g^−1^.

### 2.5 Determination of flavonoids and anthocyanin content

The content determination of flavonoids and anthocyanin was slightly modified according to Yang et al. (2019). Sample was extracted by 1% HCl-methanol solution with 20 min equilibration at 4°C under dark conditions. After centrifugation at 12,000 *g* for 10 min, the absorbance of the supernatant was measured at 325 nm, 530 nm, and 600 nm by a μQuant microplate reader (BIO-TEK, Germany), respectively. The absorbance at 325 nm represented flavonoids, and rutin was used to prepare the standard curve (*y* = 0.0079*x* + 0.0402, *R* ^2^ = 0.9989). The absorbance difference between 530 nm and 600 nm represented anthocyanin content, and peonidin was used to prepare the standard curve (*y* = 0.0522*x* + 0.009, *R* ^2^ = 0.9994).

### 2.6 Hydrogen peroxide content (H_2_O_2_), superoxide anion (O_2_^−^·) scavenging ability, CAT, and APX activities

The H_2_O_2_ content was determined using kits (AKAO009M, Boxbio, Beijing, China). The result was expressed in μmol·g^−1^. The O_2_^−^· scavenging ability (M0115A) and CAT activity (M0104A) were measured using kits (Suzhou Michy Biomedical Technology Co., Ltd., Suzhou, China).

The measurement of APX activity was modified according to Hu et al. (2012). Sample (1.0 g) was homogenized with 5.0 mL of ice-cold 0.1 mol L^−1^ potassium phosphate buffer at pH 7.5 (containing 0.1 mmol/L EDTA, 1 mmol/L ascorbic acid, and 2% PVPP) and centrifuged (12,000 *g*, 30 min, 4°C). The supernatant was collected as enzyme extraction for the determination of APX activity. The APX enzymatic reaction system included 2.6 mL of 50 mmol L^−1^ potassium phosphate buffer (containing 0.1 mmol L^−1^ EDTA and 0.5 mmol L^−1^ ascorbic acid), 0.1 mL of enzyme extraction, and 0.3 mL of 2 mmol L^−1^ H_2_O_2_ solution. One unit of enzyme activity (U) was defined as 0.01 decrease of absorbance per gram sample per minute at the wavelength of 290 nm. The APX activity was expressed as U·g^−1^. All the experiments were performed with three replications.

### 2.7 Statistical analysis

Figures were created and principal component analysis (PCA) was performed using Origin 8.0 (OriginLab Co., Northampton, MA, USA). Statistical analysis and significance analysis (independent sample *t*-test) were performed using the mean values of test results (SPSS 17.0; SPSS Inc., Chicago, IL, United States).

## 3 Results

### 3.1 Effect of SAEW treatment on weight loss control

**Figure 1:**
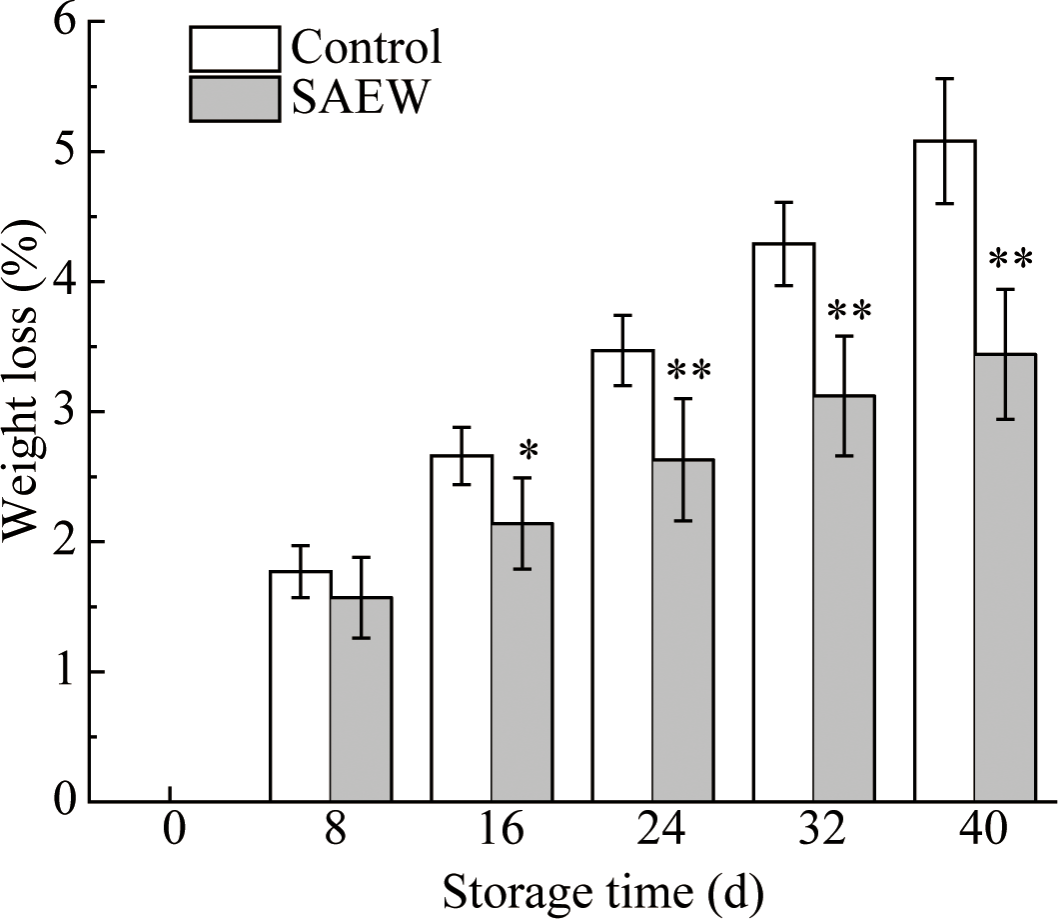
Changes of weight loss in control and SAEW-treated fresh red waxy corn during cold storage. Values are the means ± standard error (SE) (*n* = 3). Note: The asterisks at a particular storage time indicating significant difference between control and SAEW-treated samples (*p < 0.05; **p < 0.01).

The weight loss of fresh red waxy corn showed increased trend during the whole storage (**Figure 1**). It increased approximately 2% in the first 8 d compared with the initial samples in both groups. However, as the storage time extended, the weight loss of the control group increased significantly faster than the SAEW-treated group after 8 d (**Figure 1**). At the end of the storage, the highest weight loss of the control group reached 5.08% ± 0.48%, which increased approximately 47% compared with the SAEW group (**Figure 1**). The results demonstrated that SAEW treatment effectively reduced the weight loss of fresh red waxy corn compared with the control during cold storage.

### 3.2 SAEW delayed the increase of corn ear firmness

**Figure 2:**
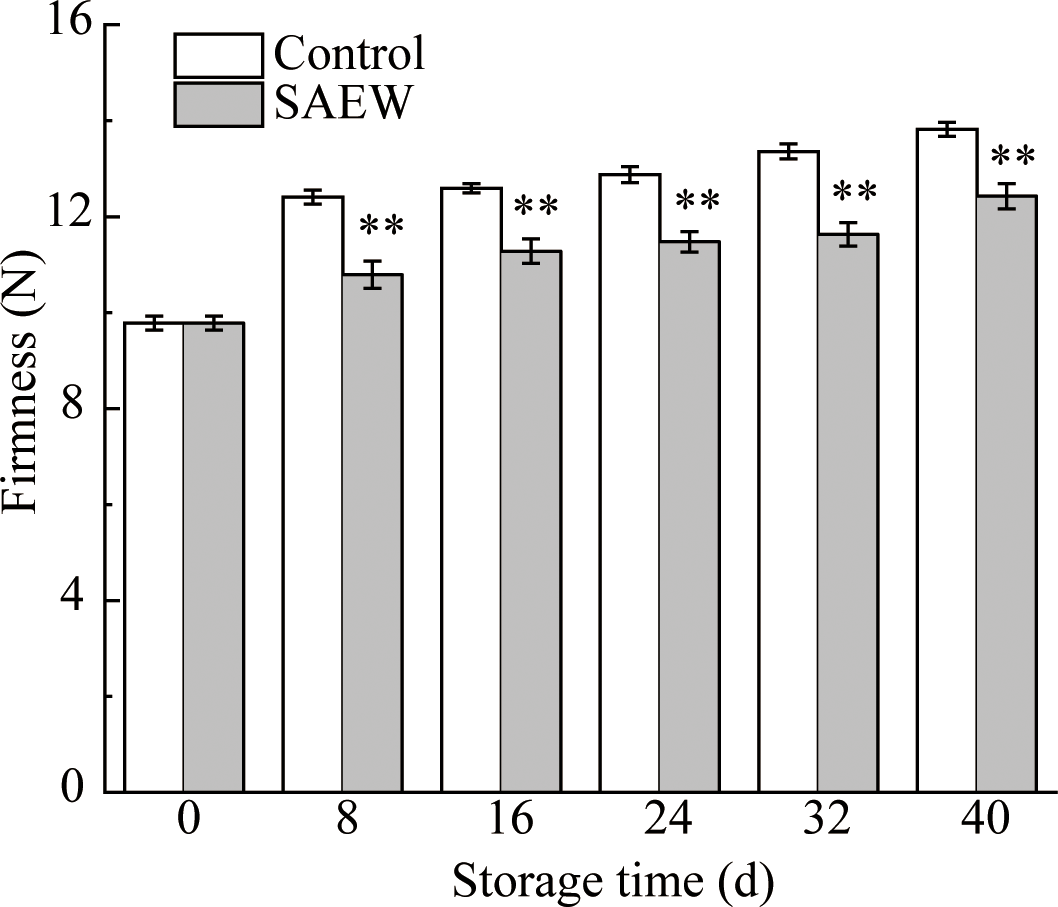
Changes of firmness in control and SAEW-treated fresh red waxy corn during cold storage. Values are the means ± SE (*n* = 6). Note: The asterisks at a particular storage time indicate significant difference between control and SAEW-treated samples (*p < 0.05; **p < 0.01).

As displayed in **Figure 2**, the firmness of fresh red waxy corn continued to increase as the storage duration extended. The firmness rapidly increased 26.8% of control samples, while the SAEW only increased 10.3%, compared to the initial samples in the first 8 d. The firmness of SAEW treatment was dramatically 10.1% lower than the control group during the whole storage duration (**Figure 2**). Even if the storage reached 40 d, the firmness of SAEW treatment was dramatically 10.1% lower than the control group. The SAEW treatment could inhibit the firmness increase of fresh red waxy corn and maintain its tenderness.

### 3.3 Characterization of the microstructure with the SEM

**Figure 3:**
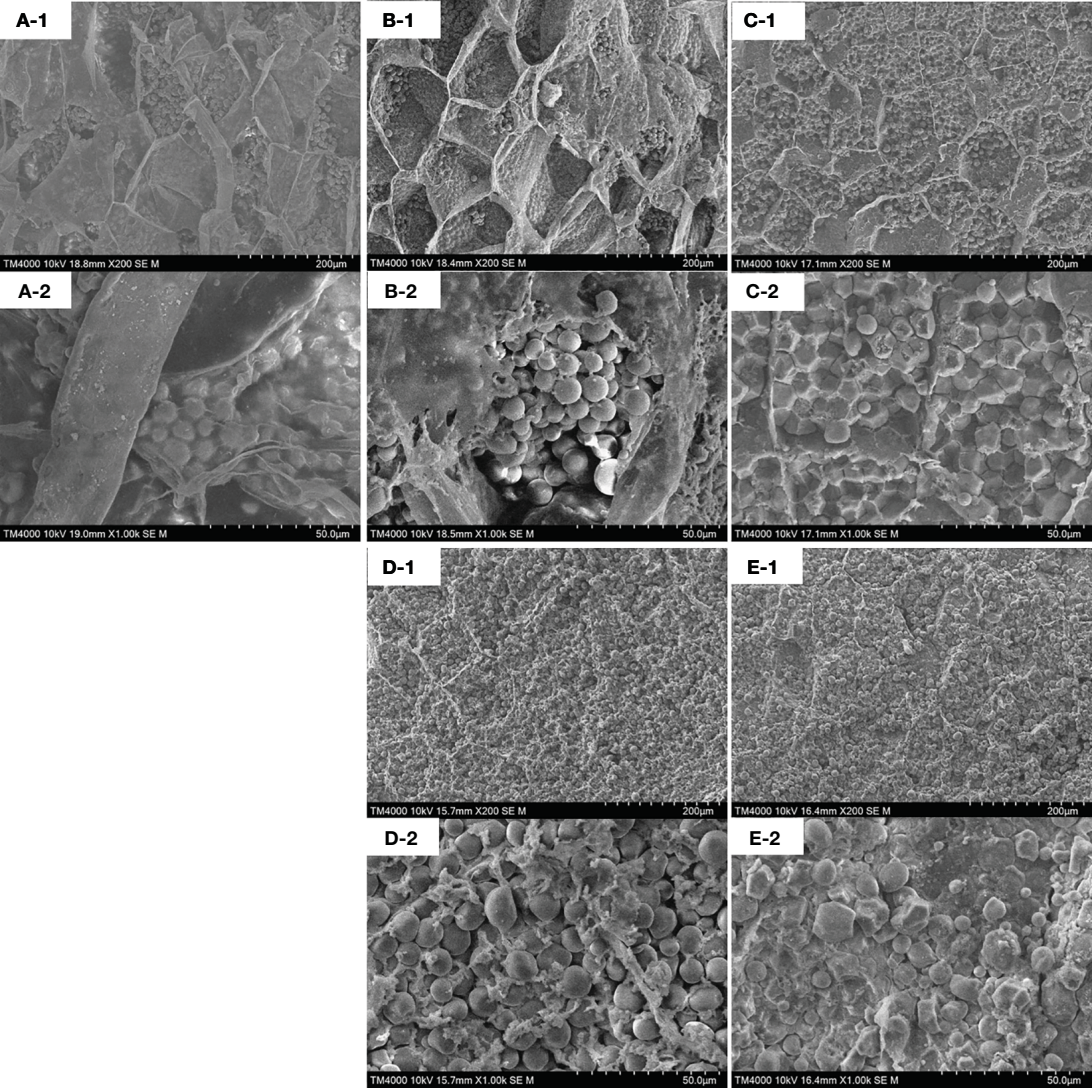
Scanning electron micrographs of control and SAEW-treated fresh red waxy corn during cold storage. (**A**, samples at day 0; **B**, SAEW-treated samples at day 8; **C**, control samples at day 8; **D**, SAEW-treated samples at day 40; **E**, control samples at day 40; **1**, ×200; **2**, ×1,000.).

In order to analyze the influence of SAEW treatment on the texture properties of the kernels of corn ears, the cross-section microstructure of the fresh red waxy corn was photographed by SEM. As shown in **Figure 3A-1**, postharvest fresh red waxy corn at day 0 had a clear and intact endosperm cell wall. **Figure 3A-2** shows that starch granules had a spherical structure and smooth surfaces, and they were loosely and regularly arranged. After 8 d, SAEW-treated samples still had an obvious intact endosperm cell wall (**Figure 3B-1**) and all the full round starch granules were orderly encapsulated in the endosperm (**Figure 3B-2**). However, the endosperm cell wall of control samples began to degrade (**Figure 3C-1**). The starch granules became wrinkled with a polygonal structure and densely packed (**Figure 3C-2**). At the end of the storage period, endosperm cell walls of SAEW-treated samples became blurred (**Figure 3D-1**), while the starch granules still had a spherical structure but much more gathered than at day 8 (**Figure 3D-2**). Compared to SAEW-treated samples, the internal structure of the control sample was seriously damaged. The endosperm cell walls were more blurred, caused by an increased number of single granules (**Figure 3E-1**). Meanwhile, the edges of the starch granules were seriously shrunk and broken, displaying an irregular bulk density (**Figure 3E-2**). These results indicated that SAEW treatment effectively maintained the normal microstructure of fresh red waxy corn kernels.

### 3.4 Effects of SAEW on the maintenance of TSS, moisture, and soluble protein content

TSS and moisture content have a great influence on the quality and taste of fresh agriculture products (Zhao et al., 2021). **Table 1** shows the TSS and moisture content of SAEW-treated and control samples during cold storage. The TSS of fresh red waxy corn continuously decreased during the whole storage period except after 40 d. The SAEW effectively delayed the TSS decline, especially for the first 8 d. The TSS decreased from 14.37% to 11.66% in control samples, with an 18.9% decline, while it only decreased to 12.71% for SAEW treatment, resulting in significant higher content than control at 8 d (*p* < 0.01). Afterwards, the decline of the TSS became slower, without obvious difference in both groups. However, the TSS content showed an increased trend from 32 d to 40 d in both groups, while the control group exhibited a higher TSS content than the SAEW group at 40 d (**Table 1**).

**Table 1:** Changes of TSS, moisture content, and soluble protein content in control and SAEW-treated fresh red waxy corn during cold storage.

Treatment	Storage time (d)	TSS (%)	Moisture content (%)	Soluble protein content (mg g^−1^)
Control	0	14.37 ± 0.36	58.05 ± 0.54	4.24 ± 0.16
	8	11.66 ± 0.07	57.79 ± 0.54	4.49 ± 0.16
	16	11.13 ± 0.07	57.87 ± 0.19	3.78 ± 0.22
	24	10.78 ± 0.20	58.25 ± 0.04	3.64 ± 0.17
	32	10.79 ± 0.06	56.71 ± 0.52	2.98 ± 0.07
	40	11.34 ± 0.09	54.25 ± 0.2	2.94 ± 0.10
SAEW	0	14.37 ± 0.36	58.05 ± 0.54	4.24 ± 0.16
	8	12.71 ± 0.02**^1^	58.44 ± 0.27	4.55 ± 0.17
	16	11.25 ± 0.21	58.2 ± 0.48	4.95 ± 0.29**
	24	11.09 ± 0.07	59.47 ± 0.65*	4.64 ± 0.04**
	32	10.83 ± 0.16	58.57 ± 0.32**	3.91 ± 0.31**
	40	11.10 ± 0.05*	58.02 ± 0.24**	3.72 ± 0.14**

^1^The asterisks at a particular storage time indicate significant difference between control and SAEW-treated samples (*p < 0.05; **p < 0.01).

The moisture content of SAEW-treated fresh red waxy corn was steady during the whole storage duration, while that of control samples maintained the status only up to 24 d, as the obvious decreased trend was observed from 32 d to 40 d (**Table 1**). The SAEW maintained a significantly higher moisture content compared to the control group from 24 d to 40 d. For example, the moisture content of control samples dramatically decreased by 6.59% compared to SAEW treatment at 40 d (**Table 1**). The trend of soluble protein content of fresh red waxy corn increased firstly and then decreased till the end of cold storage. The soluble protein content of SAEW-treated fresh red waxy corn increased up to 16 d with a highest value of 4.95 mg g^−1^, while that of control only increased to 8 d with 4.49 mg g^−1^. Moreover, the SAEW group maintained a significantly higher soluble protein content than the control group from 16 d to 40 d. Thus, SAEW treatment not only elevated the soluble protein content peak, but also decreased the decline rate during cold storage. Overall, these results indicated that SAEW treatment could inhibit the decrease of TSS, moisture, and the soluble protein content of fresh red waxy corn during cold storage.

### 3.5 Changes of the flavonoid and anthocyanin content

**Figure 4:**
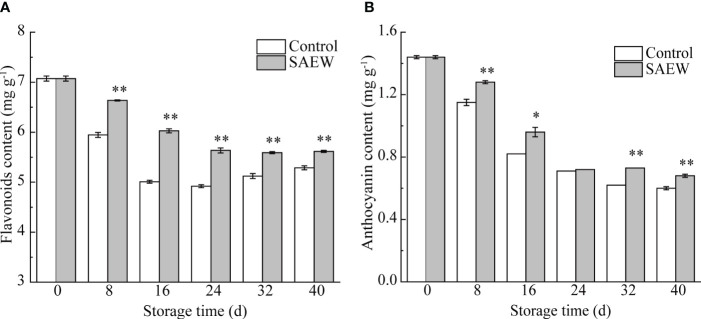
Changes of flavonoids **(A)** and anthocyanin **(B)** content in control and SAEW-treated fresh red waxy corn during cold storage. Values are the means ± SE (*n* = 3). Note: The asterisks at a particular storage time indicate significant difference between control and SAEW-treated samples (*p < 0.05; **p < 0.01).

As shown in **Figure 4A**, the flavonoid content showed a decreasing trend till day 24 compared with initial samples in both groups. However, the falling rate and extent in the SAEW group were significantly less than the control group. For instance, the flavonoid content of the control group decreased by 29.1% compared with the initial sample at day 16, while the SAEW treatment only reduced by 14.7%. Hence, the flavonoid content in the SAEW-treated samples was notably higher than those in the control during the whole storage period. Though the flavonoid content showed a slight increasing trend from day 24 to day 40 in the control group, a significantly higher concentration was observed in the SAEW-treated samples.

The anthocyanin content showed a decreasing trend during the whole storage duration in both control and SAEW samples (**Figure 4B**). Interestingly, the declining trend continued until day 32 in the control group, but only till day 24 in the SAEW group. Correspondingly, the control group maintained a dramatically lower content of anthocyanin compared with the SAEW-treated samples during the storage period except for day 24 (**Figure 4B**). These results indicated that SAEW treatment effectively delayed the decline of flavonoids and anthocyanin, which maintained the attractive appearance and good nutritional quality of the fresh red waxy corn during cold storage.

### 3.6 Effects of SAEW on metabolism of antioxidant capacity

**Figure 5:**
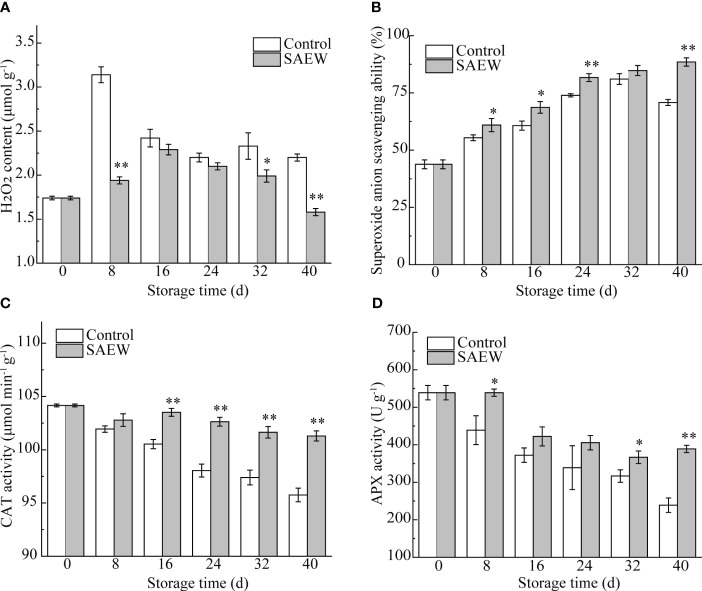
Changes of H_2_O_2_ content **(A)**, O_2_^−^· scavenging ability **(B)**, CAT activity **(C)**, and APX activity **(D)** in control and SAEW-treated fresh red waxy corn during cold storage. Values are the means ± SE (*n* = 3). Note: The asterisks at a particular storage time indicate significant difference between control and SAEW-treated samples (*p < 0.05; **p < 0.01).

H_2_O_2_ and O_2_^−^· are typical species of ROS, which would lead to oxidative damage to the cell. As shown in **Figure 5A**, the H_2_O_2_ content in control samples increased to peak at day 8 and then decreased, while the SAEW delayed the peak to day 16. Correspondingly, the cold storage caused the H_2_O_2_ content to dramatically increase from 1.74 μmol g^−1^ to a peak value of 3.14 μmol g^−1^, with an 80% elevation, while it only increased by 31.6% on day 16 compared with the initial samples in SAEW-treated samples. Interestingly, the difference of the H_2_O_2_ content between the two groups was obvious in the early and late stages during the storage. For instance, the control group showed the biggest increase of H_2_O_2_ content (61.9%) on day 8 compared with SAEW-treated samples on the same day, and the significant differences were also observed from day 32 to day 40. These results demonstrated that SAEW treatment reduced the accumulation of H_2_O_2_ content and delayed the occurrence of peak value of H_2_O_2_ content during cold storage.

The O_2_^−^ scavenging ability in both groups displayed an increased trend during cold storage except for day 40 in control samples (**Figure 5B**). The SAEW maintained a significantly higher O_2_^−^· scavenging ability than the control during the storage except for day 32. For example, the O_2_^−^· scavenging ability level of the control group only reached 78.4% of the SAEW at the end of the storage period. These results indicated that SAEW treatment could obviously elevate the O_2_^−^· scavenging ability of fresh red waxy corn.

CAT and APX are important antioxidant enzymes that can clear the excess H_2_O_2_ and alleviate the oxidation damage. As shown in **Figure 5C**, CAT enzyme activities showed a decreasing trend in the control group during the storage duration, while the SAEW treatment delayed the decreasing rate, and hence, higher enzyme activities compared with the control samples were determined from day 16 to day 40. The CAT activity decreased to 91.93% of the initial samples, but the SAEW treatment only reduced no higher than 3% on day 40. Similarly, the APX activities exhibited the same decreasing trend during the storage, and SAEW treatment maintained the enzyme activities well, especially for the early and late stage. For instance, the CAT activity of the initial sample was reduced to 81.4% on day 8 in the control group, while the SAEW group barely changed. As the storage extended to day 40, the control group declined to 44.3% compared with 0 d, while the SAEW treatment still occupied 72.2% of the initial sample, which maintained approximately 1.6-fold that of the control. These results demonstrated that SAEW treatment could highly preserve antioxidant enzymes’ activity in fresh red waxy corn.

The above findings indicated that SAEW treatment could effectively delay the fruit senescence and quality deterioration by improving the O_2_^−^· scavenging ability and antioxidant enzyme activities during the cold storage.

### 3.7 Relationships of different indexes present by PCA and correlation analysis

**Figure 6:**
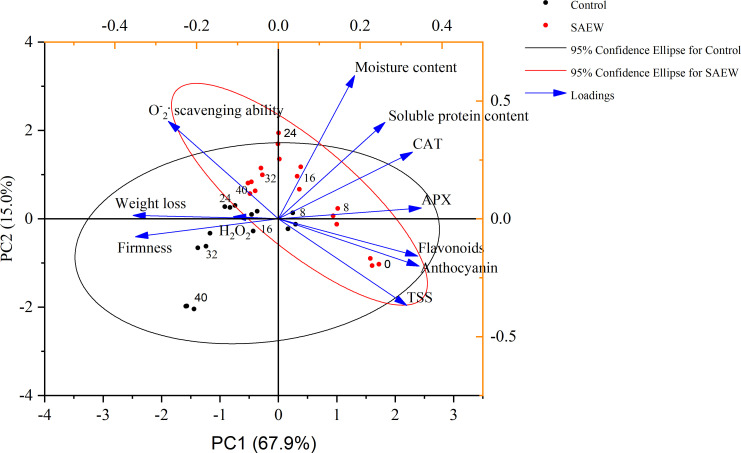
Principal component analysis score and loading plot assessing the variance of control and SAEW-treated fresh red waxy corn during cold storage.

PCA is a mathematical tool based on multivariate statistics, which could reduce the dimension and simplify the data for extracting features and revealing the relationship among different variables (Liu et al., 2018; Leng et al., 2021). As shown in **Figure 6**, the total scores of PC1 and PC2 reached 82.9%, explaining the variance of different samples well. As we can see, the 95% confidence ellipses generally reflected the variation within the group, and the smaller circle of SAEW treatment indicated the mild changes during the entire storage compared to the control. The SAEW samples were located closer to the 0 d sample than the control group, especially for the early stage of day 8 and day 16 and the late stage of day 40 in both PC1 and PC2. The PCA showed that the difference was obvious in the early stage till day 16 with a large gap in both PC1 and PC2, while the middle stage from day 24 to day 32 clustered together in both groups. Interestingly, the samples from day 24 to day 40 in SAEW were located together, while day 40 in the control group was obviously separated from the day 16 to day 32 circle, indicating the dramatic deterioration at the end of storage. In sum, the SAEW treatment samples were divided into two circles of day 8 and day 16 located at the first quadrant, and the other samples were located at the second quadrant. However, three obvious clusters were observed in the control group, day 8 located at the fourth quadrant, the day 16 to day 32 cluster located across the second and third quadrant, and day 40 located at the left bottom of the third quadrant.

The loading plot indicated the main factors that influenced the changes in varied samples. The antioxidant capacity of the samples including the antioxidant enzymes and substances clustered together with the initial samples was characterized with high TSS and soluble protein content. The SAEW samples displayed positive correlations with good characteristics. In contrast, the samples in control clustered together with some deterioration indexes, such as the weight loss, firmness, and content of H_2_O_2_. In sum, the PCA effectively distinguished and explained the variance of different samples during the cold storage.

The correlation analysis showed an accurate relationship between different indexes, indicating the key changes that influenced the quality and storage ability. The content of flavonoids and anthocyanin, and the activities of APX, CAT, and TSS showed significant positive correlations (*p* < 0.01). In contrast, the weight loss and firmness exhibited dramatic negative correlations (*p* < 0.01) with the TSS and antioxidant capacity. These results indicated the key quality index and the factors that determined the storage time.

## 4 Discussion

Fresh corn ears are healthy food loved worldwide due to their unique flavor and rich nutrients. However, corns easily deteriorate because of their high metabolism rate during postharvest storage. The quality of the corn ears was mainly influenced by the moisture content, firmness, and nutritional substances, especially flavonoids and anthocyanin, determining the antioxidant capacity. The index of weight loss was typically caused by high respiration rate and evaporation pressure during postharvest storage, resulting in the loss of the sensory quality and nutritional substance (Zhang et al., 2019; Shi et al., 2022). The weight loss reached over 5% in the control group during the storage, while the selected concentration of SAEW effectively reduced the moisture loss from day 16 till day 40, and the weight loss was maintained lower than 3.5% (**Figure 1**). The cold electrolyzed functional water (ACC 60 mg L^−1^) treatment reduced the weight loss in Hupingzao jujube during the cold storage, maintaining good sensory quality (Shi et al., 2022). The blueberries exhibited an increased firmness by preventing the high weight loss during the cold storage, which was in contrast with the fresh waxy corn. Though the acidic electrolyzed water (ACC 48 mg L^−1^) treatment had no significant influence on the weight loss of the two blueberry varieties, it significantly elevated the firmness and maintained the storage quality (Chen et al., 2017). Therefore, the fruit types and characteristics are important factors that affect weight loss and storage quality. The fresh corn is consist of bracts, corn cob, and corn kernels with large specific surface area, which are much more easily to lose the moisture and influence the quality.

The firmness was a crucial factor that determined taste quality and was influenced by the senescence extent, moisture content, and carbohydrate metabolism, especially for the structure substances of the cell wall. Given the storage time extension, the firmness increased quickly in the control group (**Figure 2**), which occurred generally during the cold storage, and similar results of firmness increasing were reported in peach, loquat, and bamboo shoots (Cai et al., 2006; Zeng et al., 2015; Liu et al., 2022). The fresh waxy corn continuously accumulated lignin after postharvest ripening and senescence, causing an increase in firmness (Gong and Chen, 2013; Gong et al., 2018). Meanwhile, the transformation from sugar to starch was a predominant metabolic process of postharvest fresh waxy corn, influencing the sensory quality of sweetness (Gong and Chen, 2013). The structure of the starch granules affected the tenderness of fresh waxy corn, and the microstructure changed gradually during the cold storage as the corn ears start to senescence (**Figure 3**). The SAEW treatment could delay the endosperm cell wall degradation and maintain the steady status of the starch granules well during the storage (**Figure 3**). Gaytán-Martínez et al. (2006) reported that the complex hierarchical structure of starch granule determined the kernel hardness and the density of the packed structure exhibited positive relationships with the high hardness value. Consistent with their research, the control samples showed a densely packed structure of starch granules along with a higher firmness (**Figure 2**). Thus, SAEW treatment maintained the structure of endosperm cell wall and starch granules, and inhibited the increase of firmness during the storage.

The quick drop of TSS was probably due to the transformation from sugar to starch during the early storage period (**Table 1**). The higher TSS of control samples stored on day 40 may due to its lower moisture content. This increased phenomenon of TSS due to the elevation of the water loss at the end of the storage was also observed in the storage of sweet cherry (Hayta and Aday, 2015). The soluble proteins were important osmotic adjustment substances and nutrients of the fresh corn ears, which increased the water retention and membrane protection capacity of the cell during accumulation. Hence, the soluble protein content was usually used as the indicator of the resistance ability of the plants to abiotic stress. The SAEW treatment significantly delayed the decline of the soluble protein content from day 16 to day 40 compared with the control group, reflecting the resistance ability to the cold storage stress (**Table 1**). Anthocyanin is the main pigment of fresh red waxy corn, and highly correlated to the appearance color of corn kernels (Wang and Stoner, 2008). The apparent color is crucial to evaluate the maturity and freshness of the fruit during storage (Gao et al., 2019). In our study, SAEW maintained a higher content of anthocyanin and flavonoids in fresh corn ears, contributing to the fresh attractive appearance at the end of the storage (**Supplementary Figure S1**). More importantly, flavonoids and anthocyanin are important bioactive compounds with high antioxidant activities, enhancing the resistance to abiotic stress. Zhang et al. (2023) reported that SAEW-treated carambola achieved better apparent quality than control by inhibiting the deterioration of chlorophyll and accumulation of carotenoids (Jiang et al., 2018; Yan et al., 2019). The SAEW treatment could retain higher contents of flavonoids, anthocyanin, and total phenolics in longan, blueberry, and carambola pulp during postharvest storage (Chen et al., 2019, 2020; Zhang et al., 2023). Therefore, it was speculated that the change in flavonoid and anthocyanin content was important for the postharvest storage of various kinds of fruits. The SAEW treatment could maintain the quality and extend the storage time by maintaining the content of the flavonoids, anthocyanin, and total phenolics, contributing much to the elevation of antioxidant capacity. Hence, the SAEW maintained higher antioxidant ability and much better nutritional quality and flavor than the control group by keeping the content of flavonoids and anthocyanin in fresh corn ears during the cold storage. For instance, the SAEW treatment contained higher content of TSS, moisture, and the soluble protein during the cold storage, influencing the sweetness and texture properties’ quality (**Table 1**).

**Figure 7:**
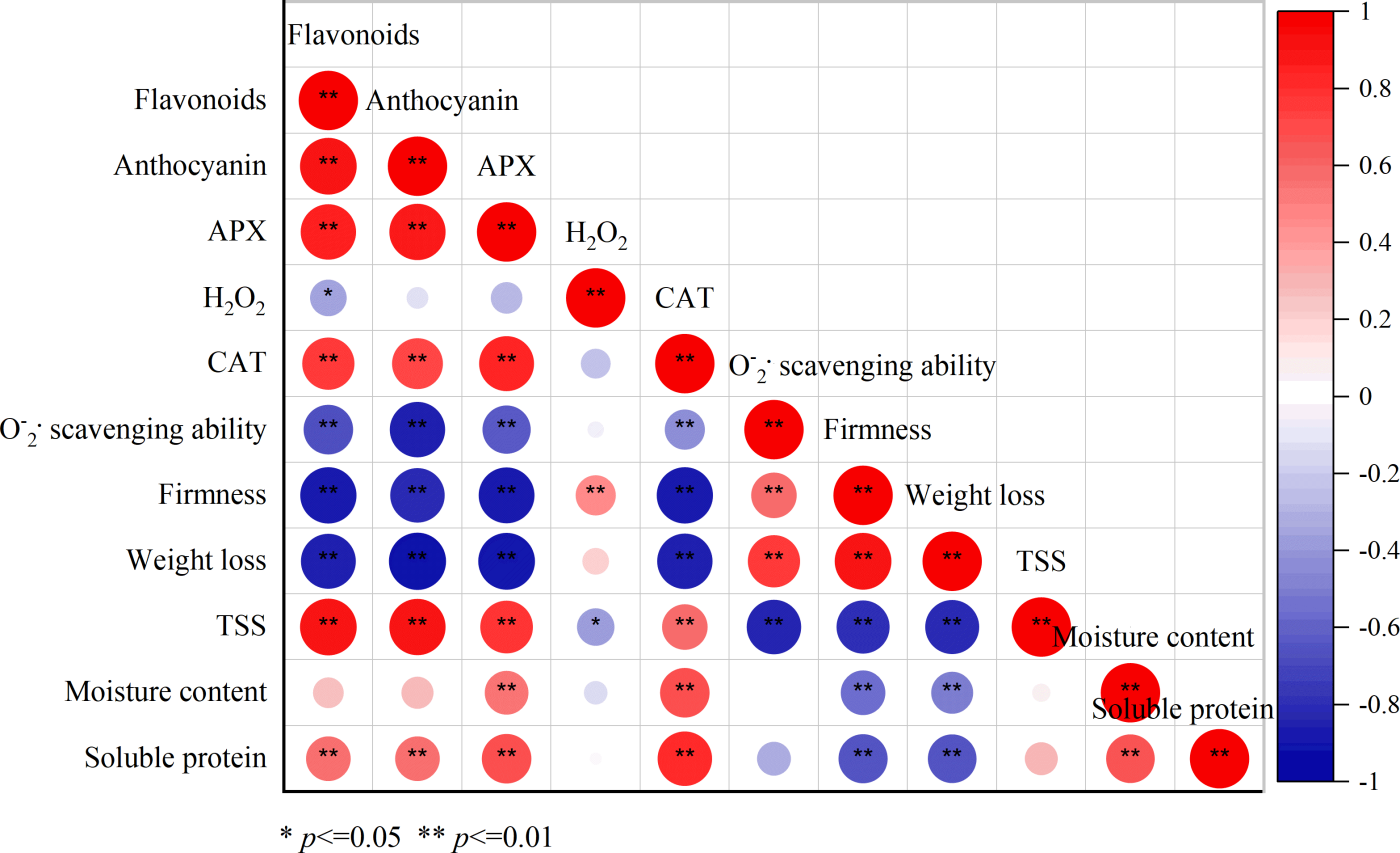
The correlation analysis of different index of both groups during the storage.

Oxidative damage was one of the main factors that caused fruit senescence and deterioration. The enzymes of superoxide dismutase, CAT, and APX are antioxidant enzymes that scavenge the excess O_2_^−.^ and H_2_O_2_, keeping the balance of ROS metabolism and alleviating the oxidation damage. Hence, the antioxidant enzyme activities and antioxidant substances were important for extending the storage duration and alleviating fruit decay (Lin et al., 2017; Chen et al., 2019). For instance, the AEW treatment inhibited fruit disease by enhancing the activities of CAT, APX, and POD during storage in longans and jujube fruit (Tang et al., 2021; Shi et al., 2022). As shown in **Figure 5**, the SAEW treatment fortified CAT, APX, and O_2_^−^· scavenging ability and decreased the production rate and peak of H_2_O_2,_ preventing the fresh red waxy corn from oxidative damage during the cold storage. Furthermore, the PCA loading plot analysis showed that the determined antioxidant enzyme, flavonoids, and anthocyanin were clustered together with the early storage stage fresh corn ears, contributing to the sensory quality by TSS, soluble protein content, and moisture metabolism (**Figure 6**). In contrast, the high H_2_O_2,_ weight loss, and firmness, causing oxidative injury and undesirable texture properties, were loaded together with the control group, especially at the end of the storage of 40 d (**Figure 6**). The PCA indicated that CAT, APX, flavonoids, anthocyanin, and TSS are the key factors influencing the storage time and quality, and the correlation analysis further revealed the significant relationship of different indexes. Consistent with the PCA, the content of flavonoids was significantly positively correlated with anthocyanin, APX, CAT, and the quality characteristics of TSS and soluble protein (*p* < 0.01). Correspondingly, the firmness, weight loss, H_2_O_2_, and the O_2_^.-^ scavenging ability displayed significant negative correlations with flavonoids (**Figure 7**). In sum, the SAEW treatment maintained sensory quality and extended the storage time by elevating the antioxidant enzyme activities and accumulation of flavonoids and anthocyanin.

## 5 Conclusion

This study investigated the effect of the selected SAEW on the nutrition quality maintenance of fresh red waxy corn and explored the preservation mechanism during cold storage. The SAEW treatment significantly suppressed the increase in weight loss and firmness, but elevated the content of TSS, moisture, and soluble protein. Furthermore, the SAEW treatment prevented the overproduction of H_2_O_2_ via enhancing O_2_^−^· scavenging ability and maintaining higher antioxidant enzyme activity including CAT and APX. In addition, the decline of the flavonoids and anthocyanin was delayed by SAEW compared with the control, which contributed to both the nutritional quality and antioxidant capacity of the fresh corn ears during the cold storage. The PCA and correlation analysis comprehensively demonstrated that SAEW treatment effectively preserved the quality of fresh red waxy corn during storage by maintaining the antioxidant enzyme activities and the accumulation of antioxidant compounds. This study indicated that SAEW treatment could be a promising approach for maintaining quality and prolonging the shelf life of harvested fresh red waxy corns. However, the mechanism of the SAEW preservation effect is necessary to further analyze the relationships between firmness and lignin metabolism.

## Data availability statement

The original contributions presented in the study are included in the article/**Supplementary Material**. Further inquiries can be directed to the corresponding author.

## Author contributions

CW: Writing – original draft, Writing – review & editing, Conceptualization, Methodology. HL: Formal analysis, Writing – review & editing, Validation. CL: Methodology, Writing – review & editing. YW: Software, Writing – review & editing. JW: Data curation, Investigation, Writing – original draft. YZ: Formal analysis, Writing – review & editing. XW: Formal analysis, Writing – review & editing. BC: Formal analysis, Writing – review & editing. WY: Funding acquisition, Writing – review & editing. YQ: Funding acquisition, Writing – review & editing.

## Supplementary material

Supplementary Figure 1 Photographs of control and SAEW-treated fresh red waxy corn during cold storage.
