# Supplementary figures and images for: Effects of slightly acidic electrolyzed water on the quality and antioxidant capacity of fresh red waxy corn during postharvest cold storage
Source: Front Plant Sci. 2024 Jun 13;15:1428394. doi: 10.3389/fpls.2024.1428394 (PMC11208686; doi:10.3389/fpls.2024.1428394)

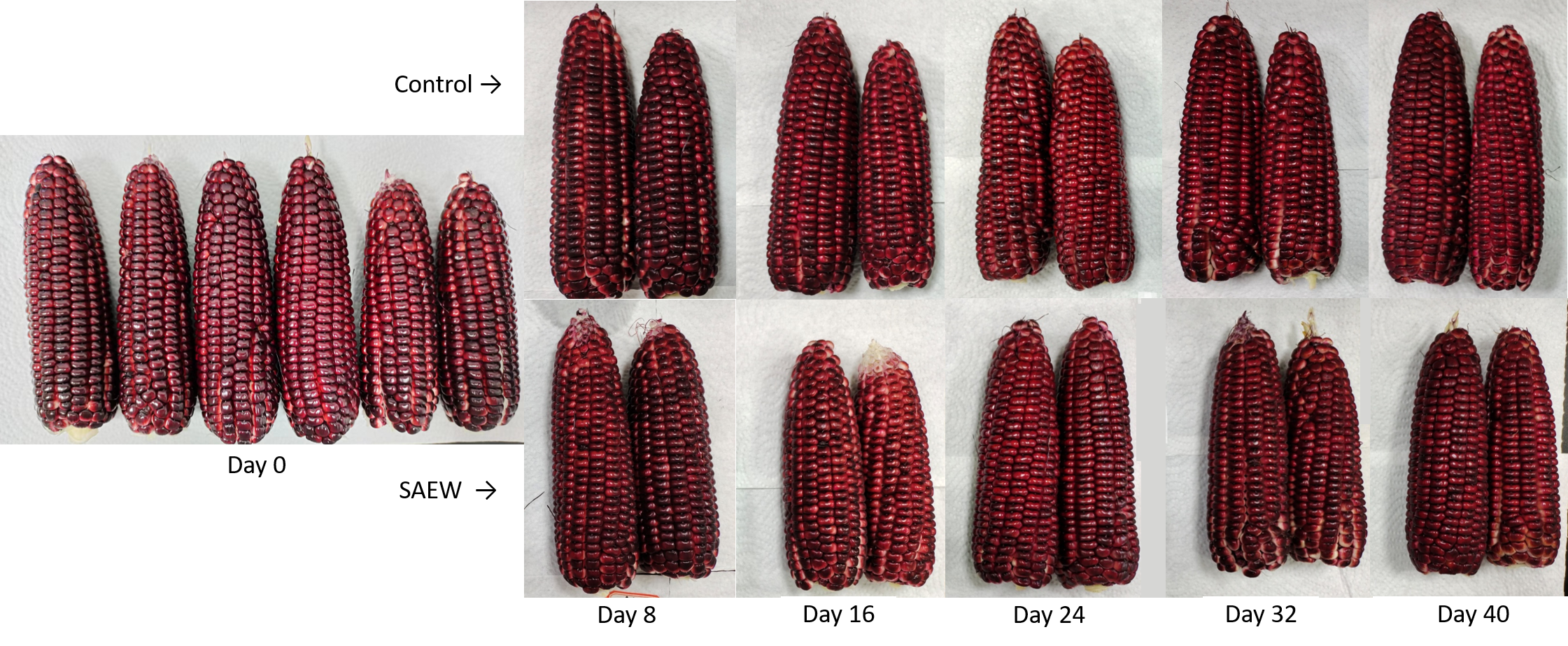

Supplement: Supplementary Figure 1 — Photographs of control and SAEW-treated fresh red waxy corn during cold storage. [file Image_1.tif]
